# A novel method for the quantification of industrial and municipal waste materials for environmental hazard assessment

**DOI:** 10.1016/j.mex.2020.101182

**Published:** 2020-12-16

**Authors:** Mohd Hariri Arifin, John Stephen Kayode, Muhammad Khairel Izzuan Ismail, Abdul Manan Abdullah, Asha Embrandiri, Nor Shahidah Mohd Nazer, Azrin Azmi

**Affiliations:** a Program Geologi, Pusat Sains Bumi dan Alam Sekitar, Fakulti Sains dan Teknologi, Universiti Kebangsaan Malaysia, 43600 Bangi, Selangor, Malaysia; b Universiti Teknologi PETRONAS, Institute of Hydrocarbon Recovery, Department of Research and Innovations. Shale Gas Research Group, Persiaran UTP, 32610 Seri Iskandar, Perak Darul Ridzuan, Malaysia; c Geo Technology Resources SDN BHD. 31-1, Jalan Mawar 5B, Taman Mawar, 43900 Sepang, Selangor; d Department of Environmental Health, College of Medicine and Health Sciences, Wollo University. Dessie P.O. Box 1145, Amhara

**Keywords:** Evaluation of environmental hazardous materials, Industrial and municipal wastes plumes, Kepong, Kuala Lumpur, Peninsula Malaysia, Quantification of depth and volume of the contaminant plumes

## Abstract

A novel methodological approach was developed to quantified the volume of industrial waste desposal (IWD) site, combined with municipal waste materials (MWM), through the integration of a non-invasive, fast, and less expenssive RES2-D Electrical Resistivity Technique (ERT), using Wenner-Schlumberger electrode array geophysical method with Oasis Montaj software. Underground water bearing structures, and the eco-system are being contaminated through seepage of the plumes emanating from the mixtures of the industrial waste materials (IWM), made of moist cemented soil with municipal solid wastes (MSW) dumped at the site. The distribution of the contiminant hazardous plumes emanating from the waste materials' mixtures within the subsurface structural lithological layers was clearly map and delineated within the near-surface structures, using the triplicate technique to collect samples of the soil with the waste mixtures, and the water analysis for the presence of dissolved ions. The deployed method helped to monitor the seepage of the contaminant leachate plumes to the groundwater aquifer units via the ground surface, through the subsurface stratum lithological layers, and hence, estimation of the waste materials' volume was possibly approximated to be 312,000 m^3^.

In summary, the novel method adopted are as presented below:

• The novel method is transferable, reproduce-able, and most importantly, it is unambiguous technique for the quantification of environmental, industrial and municipal waste materials.
• It helps to map the distribution of the plumes emanating from the waste materials' mixtures within the subsurface structural lithological layers that was clearly delineated within the near-surface structures underlain the study site.
• The procedure helped in the monitoring of leachate contaminants plumes seepages into the surface water bodies and the groundwater aquifer units, via the ground surface, through to the porous subsurface stratum lithological layers.

## Highlights

• Development of a novel method to assess environmnetal hazards of industrial, and municipal wastes.
• Application of the inverted RES2-D data using the Oasis Montaj to generate a rectangular prism model.
• Using the rectangular prism model developed to estimate the volume of IWM and MSW materials.
• Quantification of the leachate contaminant plumes flow from IWM and MSW for remediation.

## Graphical abstract

**Figure:**
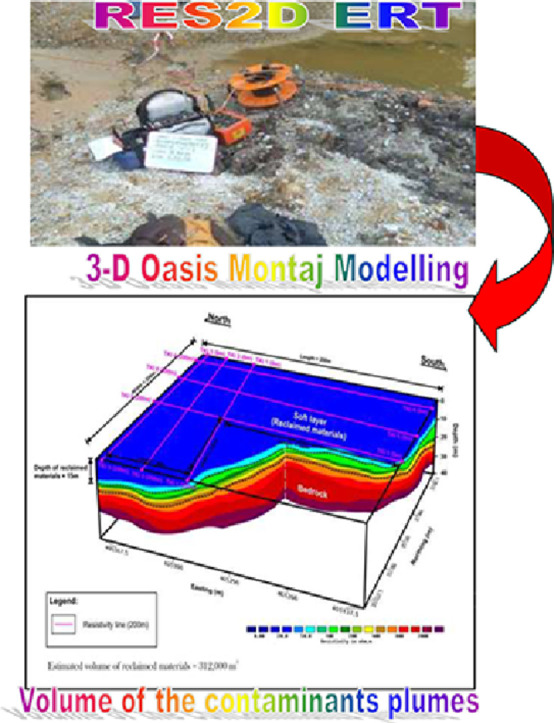

Specifications table

**Table:** 

Subject Area	Geophysics, Municipal Engineering and Urban Design
More specific subject area	Waste Management and Disposal
Method name	2-Dimensional Electrical Resistivity Technique (ERT) geophysical method integrated with Oasis Montaj software.
Name and reference of original method	The RES2DINV inverse modelling software from Loke (2016) [2], for the Two-dimensional model of the subsurface lithological resistivity layers were customized, to generate RES2-D pseudo-sections which characterize a bi-dimensional model of the subsurface stratum underlain the study site. The distance against the estimated vertical depths variations obtained from the geoelectrical inverted resistivity values recorded were plotted. The inverted RES2D ERT recorded, together with the GPS readings for the coordinates, and elevations of each electrode positions along the geophysical survey lines were integrated together to generate the 3-D model applied in the Oasis Montaj Software (2014) [3]. The output 3-D model produced from the Oasis Montaj Software helped to quantify the approximate contaminants plumes' volume from the prism shape developed. 2. Loke M.H. 2016. Tutorial on: 2-D and 3-D electrical imaging surveys. http://www.geotomosoft.com. 3. Geosoft®, *Oasis Montaj Data Processing and Analysis (DPA) System for Earth Science Applications.* ver. 8.3. Toronto, Ontario. Geosoft Inc., 2014.
Resource availability	The main research and data articles are accessible at; a. For the main article, https://doi.org/10.1016/j.jhazmat.2020.124282 b. For the data article, https://doi.org/10.1016/j.dib.2020.106595

## *Method details

**Fig. 1:**
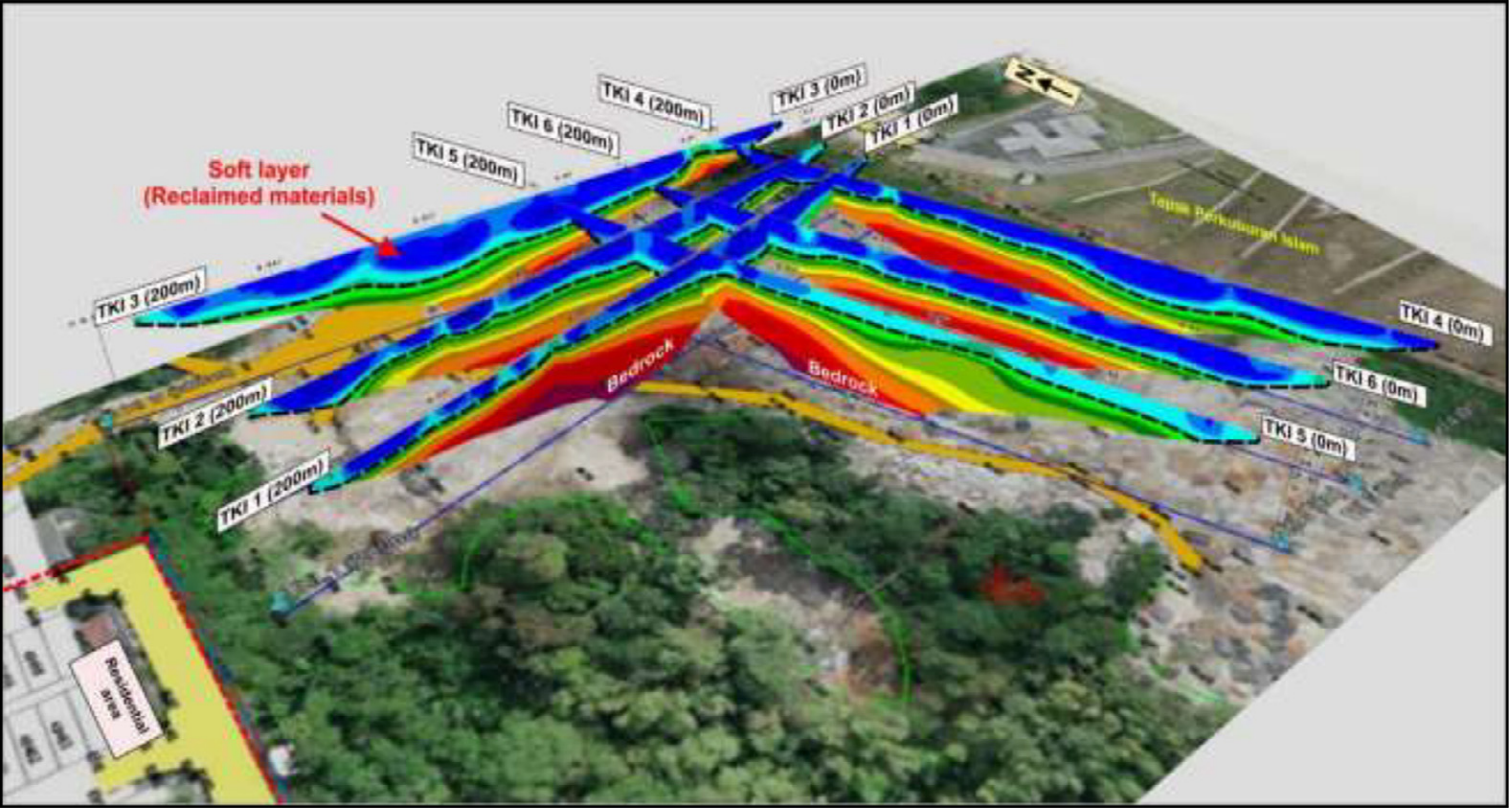
Plotted RES2D ERT profiles evenly distributed across the study site to adequately cover the waste materials, modified from [1].

**Fig. 2:**
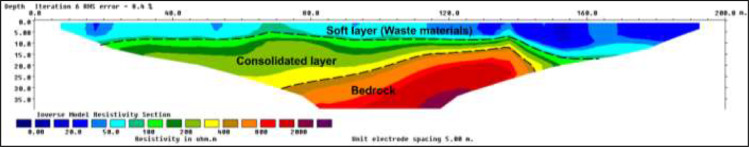
A typical RES2D ERT geophysical survey profile data with clear demarcation of the subsurface lithological layers as ploted along the survey line modified from [1].

The method deployed for recording subsurface parameters from RES2D geophysical survey of the area was adequately distributed within the subsurface stratum as presented in Fig. 1. The detailed recorded depth to the contaminant plumes, ranged between 10 and 15 m, while the corresponding resistivity distributions data from between about 0-100 Ohm-m as the waste materials. The saturated and unsaturated strata comprises of the consolidated zone together with the groundwater aquifer units enclosed in the second layers with depths of 15 ≤ 20 m, and the corresponding resistivity distributions of between 100 ≤ 400 Ohm-m. The bedrock layer depth varied between 20 ≤ 35 m as delineated, with the corresponding resistivity values of between 400 ≤ 2000 Ohm-m. Fig. 2, showed a typical RES2D ERT geophysical survey profile with clear demarcation of the subsurface lithologic layers categorizes into three major zones, as represented by the colour codes, and plotted along each of the six survey lines,(i.e., Lines 1-3, along the E-W directions, and lines 4-6, along the N-S directions) [1,4].

## Estimation of the volume for mixtures of IWM and MSW calculated from the RES2D ERT

**Fig. 3:**
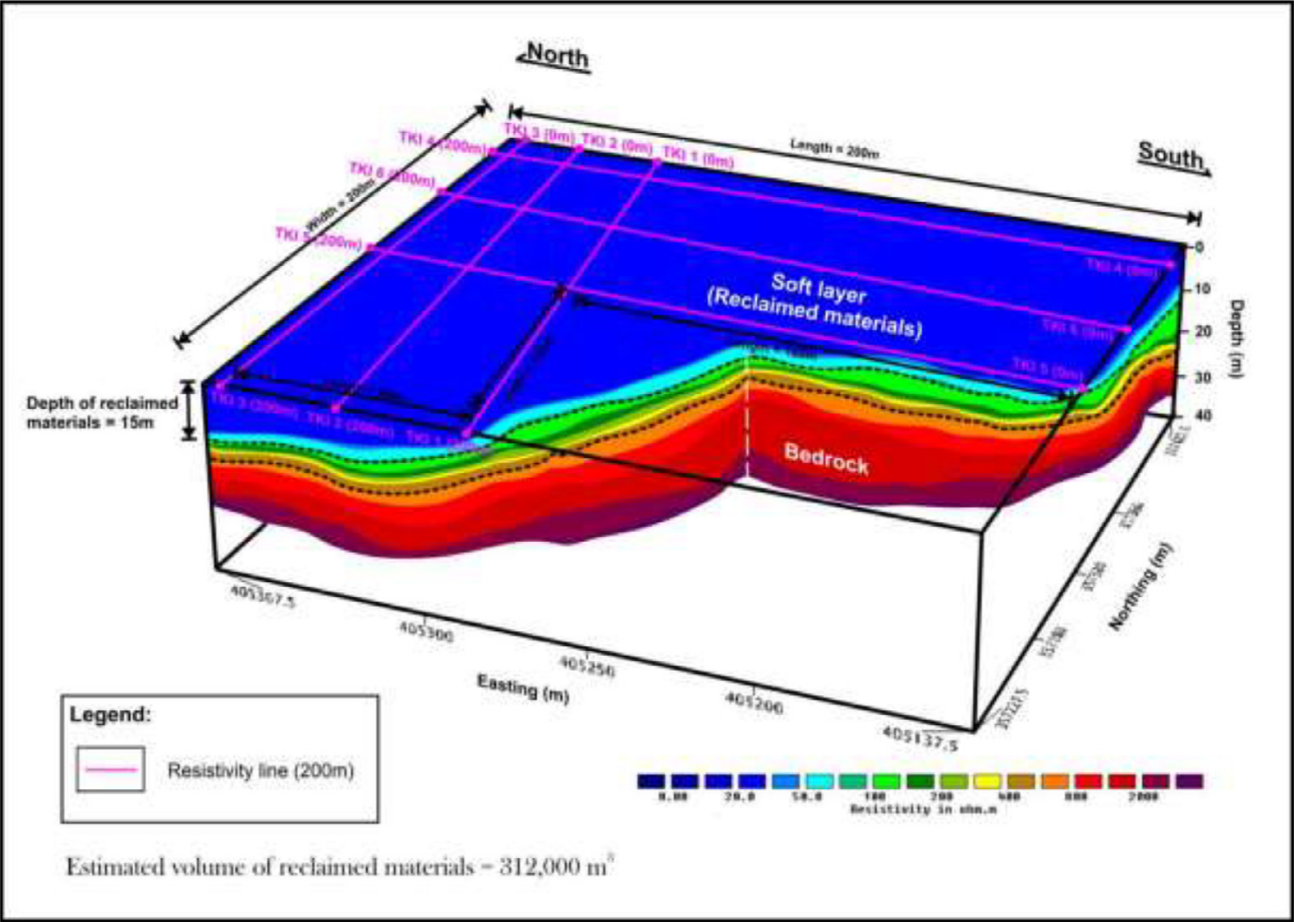
Estimated volume of leachate plumes emanating from the mixtures of IWM, and the MSW, in the Kepong area computed from the ERT, and 3-D Oasis Montaj model modified from [1].

**Fig. 4:**
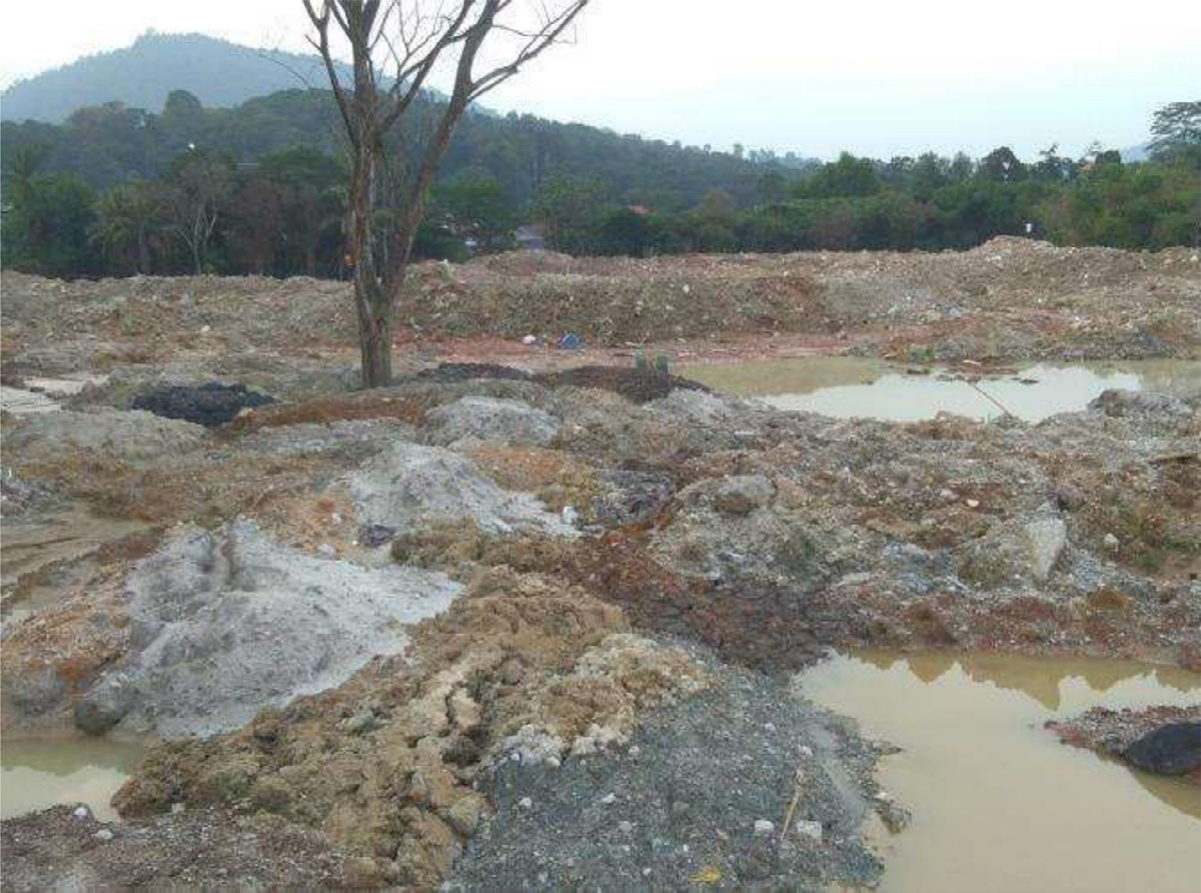
A typical view of the contaminant leachate plumes emanating from the mixtures of IWM, and the MSW materials, in the Kepong area, as captured during the RES2D ERT geoelectrical data acquisition [4].

The distributions of the subsurface lithologic layers' depth recorded corresponding to the resistivity distributions was used to quantify volume of the waste materials by means of a rectangular prism model generated using the 3-D Oasis Montaj technique, (i.e., taken the length of the geophysical survey lines χ by the depths to the contaminant plumes as the height χ by the lateral spread of the plumes as the width, with all units in m). The process was accomplished through estimation of the volume covered by the survey area, (i.e., the six survey lines formed into an L- shaped standard prism model, as indicated by the purple lines), as shown in Fig. 3, determined by initially computing the volume of the complete rectangular prism model, and take off the volume of uncover areas as indicated by the thick black rectangular solid line shown in Fig. 3. The projected volume of the contaminant leachate plumes emanating from the mixtures of industrial waste materials (IWM), and the municipal solid wastes (MSW), in the Kepong area, was approximated to be about 312,000 m^3^ taken the approximate depth to the contaminant plumes as the height to be 15 m [1,4]. The conditions of potential environmental hazard risks to human health, the ecosystems and subsurface structural features as clear evidence by the presence of some forms of physical flow of contaminant plumes, (i.e., Fig. 4), observed from the ground surface at the study site by means of effortlessly measured surface indicators designed to improve the overall quality of the 3-D image produced [5].

The study was undertaken due to the harmful effects of the dissolved ions emanated from the hazardous materials deposited at the site on the ecosystems, the environment, and human lives. Most importantly, the growing population around the dumpsite area calls for urgent action. Assessment of the novel research work of this magnitude, on IWM and MSW is most essential to the determinations of the characteristics of these hazardous ions and the movement of contaminant plumes within the subsurface strata which houses the groundwater bodies [6], [7], [8], [9]. Knowledge of the geophysical parameters and how these contaminant plumes interacts with the nature, particularly the subsurface structural lithological units motivated the design of this approach that could be replicated in any environmental conditions.

A closed observation of the huge number of different research methodologies that have been reported in literature on the environmental wastes, management of hazardous contaminant plumes, monitoring of leachable contaminants, delineation of zones susceptible to potential environmental hazards (PEH), and estimation and quantification of the contaminant plumes flows, and risks to human and ecosystems e.g., [10], [11], [12], [13], [14], showed that the present novel method invented for the purpose of quantification of the IWM and the MSW materials have not been reported.

## Results and discussion

Results from the method deployed to acquire the RES2D ERT geophysical survey, recorded along the six profiles evenly distributed across the study site, together with the GPS readings for each electrode position, were integrated together using the 3-D Oasis Montaj Software that helped in clear demarcation of the subsurface lithological layers as shown in Fig. 2 [1,4].

The novelty of this work lies on the capability of integrated geophysical evaluation of the subsurface depths, and accurate quantification of the municipal, and industrial waste materials within the study area, with the invented 3-D standard rectangular prism. The method deployed for the study is faster and cost effective. The study is significant to the discontinuation, and prevention, of potential environmental hazards, and threats to human, environmental and the ecosystems around the study site, due to the pollutants fumes from the leachate plumes flowing from the mixtures of industrial and municipal waste materials. Knowledge of the hazards associated with landfills contaminants plumes is very relevant to safety of lives, the ecosystems and subsurface structural features. It is note worthy to consider the effects of these hazardous elements no matter how meager their quantity could be. The devastating long-term health effects are not to be permitted in the society [1,4].

In the prescribed geochemical analysis of the collected soil and water samples that enclosed the mixtures of the IWM and MSW, values of the various hazardous dissolved ions were acquired using the triplicate technique to support the findings from the geoelectrical tools deployed to delineates the zones of the contaminant plumes within the subsurface lithological units. The novel method invented to study the hazards associated with landfills contaminants plumes effect on the ecosystems, and threats to human, environmental and subsurface structural features underlain the dump site area, confirmed the presence of these potential environmental hazardous dissolved ions, except for the recorded values of the Mercury presence in the soil samples that was below the detected level (bdl) [1].

The technique for geochemical analysis and assessment of the soil and water samples collected at the study site, followed the laid down world standard provided for, by the 23rd Edition of Waste Water, published in 2017. Determinations of the samples' pH used the HACH Standard Method 8000, with DR 3900 VIS Spectral Photometer, used for the Chemical Oxygen Demand (COD) analysis, certified by the Malaysian Industrial Standard, MS ISO 17025 at an accredited laboratory, Fakulti Sains dan Teknologi, Universiti Kebangsaan Malaysia, 43600 Bangi, Selangor, Malaysia [1].

The results as reported in the main article, e.g.,[1], showed that the mixtures of concentrated contaminant waste plumes from the IWM and the MSW materials create the presence of major ions of the heavy metals in the likes of; Arsenic (As; in soil samples, KS1 = 31.85, KS2 = 89.21, KS3 = 17.33 in mg/kg. In water samples, KW1 = 24.74, KW2 = 22.25, KW3 = 25.78 in µg/L), Cadmium (Cd; KS1 = 1.71, KS2 = 2.13, KS3 = 0.69 in mg/kg. In water samples, KW1 = 0.01, KW2 = 0.12, KW3 = 0.32 in µg/L), Chromium (Cr; in soil samples, KS1 = 46.39, KS2 = 15.97, KS3 = 6.21, in mg/kg. In water samples, KW1 = 15.28, KW2 = 19.42, KW3 = 11.20 in µg/L), Cobalt (Co; in soil samples, KS1 =15.97, KS2 =15.75, KS3 = 5.26 in mg/kg. In water samples, KW1 = 0.18, KW2 = 1.53, KW3 = 0.63 in µg/L), Copper (Cu; in soil samples, KS1 = 53.66, KS2 = 45.65, KS3 = 18.55 in mg/kg. In water samples, KW1 = 0.30, KW2 = 3.79, KW3 = 5.29 in µg/L), Lead (Pb; in soil samples, KS1 = 32.91, KS2 = 28.77, KS3 = 16.74 in mg/kg. In water samples, KW1 = 0.01, KW2 = 0.91, KW3 = 0.19 in µg/L), Mercury (Hg; in soil samples, KS1 = bdl, KS2 = bdl, KS3 = bdl in mg/kg. In water samples, KW1 = 0.08, KW2 = 0.09, KW3 = 0.03 in µg/L), Nickel (Ni; in soil samples, KS1 = 57.35, KS2 = 18.78, KS3 = 12.81 in mg/kg. In water samples, KW1 = 2.81, KW2 = 13.25, KW3 = 14.31 in µg/L), Zinc (Zn; in soil samples, KS1 = 119.86, KS2 = 111.77, KS3 = 35.77 in mg/kg. In water samples, KW1 = 1.47, KW2 = 6.81, KW3 = 2.69 in µg/L), and Silica, (Si; In water samples, KW1 = 5200.00, KW2 = 17210.00, KW3 = 13560.00 µg/L), could posed serious environmental and health issues as potential sources of pollution as these values recorded are well above the permissible values.

A generated standard rectangular prism shape block model of the subsurface geophysical characteristics incorporated into the geological situation of the study area are produced with the aid of the 3-D Oasis Motaj modelling allows the quantification of the contaminant plumes' volume presented in Fig. 3 and modified after [1].

## Conclusion

The invented novel methods adopted for the generation and quantification of leachate contaminant plumes in the forms of a standard rectangular prism shape block model of the subsurface geophysical characteristics, present a widespread guide for the rapid implementation in any part of the world irrespective of the terrain. The soil and water samples were collected at the same spot with known standardization that uses the triplicate technique of sample collections. Considering the economic gains from the novel method, this makes the novel method for leachate contaminant plumes quantifications less stressful, time saving, and does not require huge financial costs in comparison with other known traditional methods, e.g., the use of borehole wells. However, other methodological concerns for intending future users is in the technical know-how of the RES2DINV and the Oasis Montaj softwares that were deployed to quantify the contaminant plumes from leachate flows approximated to be about 312,000 m^3^.

## Declaration of Competing Interest

The authors declare that they have no known competing financial interests or personal relationships that could have appeared to influence the work reported in this paper.
